# Serum Level of MMP-3 and MMP-9 in Patients with Diabetes Mellitus Type 2 Infected with Epstein-Barr Virus

**DOI:** 10.3390/ijms232113599

**Published:** 2022-11-06

**Authors:** Ewa Stępień, Jakub Dworzański, Anna Dworzańska, Bartłomiej Drop, Małgorzata Polz-Dacewicz

**Affiliations:** 1 Department of Virology with SARS Laboratory, Medical University of Lublin, 20-093 Lublin, Poland; 2 Masovian Specialist Hospital, 26-617 Radom, Poland; 3 Department of Computer Science and Medical Statistics with the e-Health Laboratory, 20-090 Lublin, Poland

**Keywords:** diabetes mellitus type 2, EBV, MMP-3, MMP-9

## Abstract

Diabetes mellitus type 2 (DM2) has recently become one of the most important health problems in the world. Patients with DM2 with long-term glycaemia are more likely to become infected than the healthy population. Matrix metalloproteinases (MMPs) play a key role in tissue remodeling during various physiological processes. However, it has been reported that certain MMPs are overexpressed during the development of various human diseases. In this study, we analyzed the levels of MMP-3 and MMP-9 in the serum of DM2 patients with and without Epstein-Barr virus (EBV) infection. The study included 115 patients with DM2 hospitalized in the Internal Ward of the Masovian Specialist Hospital in Radom, Poland, who were divided into two groups: EBV-positive and EBV-negative. The levels of MMP-3 and MMP-9 were tested in the serum of patients using the ELISA method, while the presence of EBV in saliva was tested by polymerase chain reaction (PCR). The presented studies showed a significant difference in the concentration of both MMPs in diabetic patients additionally infected with EBV compared to the group of non-infected individuals. It seems that MMPs may be useful biomarkers in the diagnosis, prognosis, and monitoring of diabetes associated with EBV infection.

## 1. Introduction

Diabetes mellitus type 2 (DM2) has recently become one of the most important global health problems. It reached epidemic proportions in the year 2015, when there were nearly 415 million people with diagnosed DM2; by the year 2040 this number is expected to increase to 642 million [1,2]. According to the American Diabetes Association (ADA) definition, diabetes is a complex and chronic disease requiring constant medical care with the necessity of reducing multifactorial risks beyond glycemic control [3].

Epstein-Barr virus (EBV), belonging to Herpesviridae family, infects more than 90% of the world’s population, establishes a persistent infection and reactivates periodically into the lytic cycle [4,5,6].

Persistent infections involving various viruses affect the overall immunity of the patient. In our previous study, EBV DNA was the most prevalent in the group of diabetic patients (35.9%) and the prevalence of infections depended on the duration of the disease [7]. According to some research, patients with DM2 are more susceptible to infections due to increased virulence of various microbes under hyperglycemia [8].

Matrix metalloproteinases (MMPs) belong to a large family of zinc-dependent extracellular matrix (ECM) remodeling endopeptidases [9]. They show apparent differences in substrate specificity, cellular and tissue localization, membrane binding, and regulation. MMPs are divided into 7 groups: collagenases, gelatinases, metalloelastases, stromelysins, matrilysins, membrane-type MMPs (MT-MMPs), and other MMPs [10]. Some literature also suggests the division of MMPs into 8 groups, according to structural similarities and function: minimal domain MMPs (MMP-7 and MMP-26), simple hemopexin MMPs (e.g., MMP-3, MMP-10), gelatin binding MMPs (MMP-2 and MMP-9), furin-activated MMPs (MMP-11 and MMP-28), vitronectin-activated MMPs–MMP-21, transmembrane MMPs (e.g., MMP-14, MMP-15), GPI-anchored MMPs (MMP-17 and MMP-25), and type II transmembrane MMPs–MMP-22 [11,12]. These MMPs participate in different biological and physiological processes that are regulated by hormones, growth factors, and cytokines [13].

MMPs play a pivotal role in tissue remodeling during various physiological processes, such as embryogenesis, morphogenesis, angiogenesis, and wound repair [9]. The activity of MMPs is involved in processes that include adhesion, cell proliferation, and migration and/or apoptosis by causing the cutting of bioactive molecules that modulate these processes [10].

However, it has been described that during the development of different human diseases some MMPs are overexpressed in specific tissues [9]. Abnormal regulation of MMPs has a relevant role in pathological conditions, including inflammation, tissue destruction, infection, and host defense [9,14]. The activity of MMPs is influenced by the presence of tissue metalloproteinase inhibitors (TIMP) [15,16]. MMPs and their TIMP are essential for cell migration and tissue remodeling in both physiological and pathological states [10,17].

MMPs are among the factors that may contribute to diabetes and are involved in the pathogenesis of associated vascular changes and complications. In patients with DM2, MMPs also play an important role in the development and severity of vascular complications [18].

MMP-3—also called stromelysin-1—degrades, among others, collagen type III, IV, IX, X, fibronectin and laminin. It is involved in breaking down tight junctions via E-cadherin. It promotes a process related to changes in epithelial cells that allow them to migrate through the basal membrane [19,20,21]. MMP-3 expression increases during the activation of EBV viral infection. Researchers proved that the EBV transcription factor Zta plays the role of a transactivator necessary in the transition of EBV from latency to lytic cycle. Zta contributes to the induction of MMP-3 expression, migration, and invasion of cells infected with EBV. The EBV virus, by producing Zta and EBV latent membrane protein 1 (LMP-1), the principal EBV oncoprotein, increases the expression of MMPs. It leads to disruption of the basal membranes of cells, which promotes the development of infection [22].

MMP-9—also called gelatinase B—breaks down type I and V gelatin, type IV and V collagen, and fibronectin. MMP-9 is expressed by endothelial cells, osteoclasts, chondrocytes, osteoblasts, and tumor cells [19,20,21]. MMP-9 expression correlates with EBV infection and the form of EBV latency (type III) in which all EBV proteins needed for cell transformation, e.g., LMP-1, are expressed. LMP-1 enhances the expression of MMP-9 [23].

MMP-3 and MMP-9 are overexpressed in DM2 altering re-epithelization, tissue and extracellular matrix damage and micro- and macrovascular remodeling [9]. Metalloproteinases, e.g., MMP-9 may be useful diagnostic biomarkers for the assessment of diabetes and related disorders [9]. In diabetic patients, constant hyperglycemia induces expression and activity of MMP-9 [24]. Researchers assessed that MMP-3 and MMP-9 were co-regulated by Zta in a similar mechanism. Moreover, MMP-3 and MMP-9 induced by Zta differently influenced cell migration, but synergistically contributed to cell invasion [22].

Therefore, in this study, we analyzed the serum level of MMP-3 and MMP-9 in patients with DM2 with and without EBV infection. In addition, we were interested in the level of MMP-3 and MMP-9 in patients with DM2 with EBV infection with different durations of diabetes and body mass index (BMI).

## 2. Results

Epidemiological characteristics of EBV-positive and EBV-negative patients with DM2 are presented in Table 1. These groups were properly selected in terms of sex, age, place of residence, smoking, alcohol consumption, BMI, and duration of diabetes. In terms of sociodemographic features, smoking, alcohol consumption, BMI and duration of diabetes these groups did not differ (*p* > 0.05), so these features did not affect the values of the studied parameters. The duration of diabetes in our study is the time from the moment a patient is diagnosed with diabetes. Duration was also categorized as 1–5 years, 6–10 years, and >10 years (Table 1).

**Figure 1:**
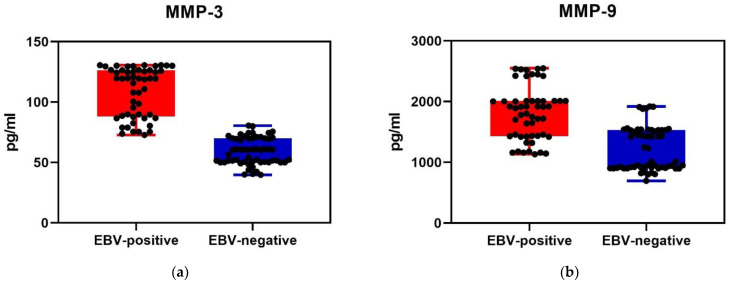
Serum level of MMP-3 (**a**) and MMP-9 (**b**) in EBV-positive and EBV-negative diabetic patients (Mann–Whitney U Test (**a**) Z = 8864; *p* < 0.001, (**b**) Z = 6188; *p* < 0.001).

Statistical analysis showed that in the EBV-positive group the values of MMP-3 and MMP-9 were significantly higher. The level of MMP-3 was lower compared to the level of MMP-9 in both groups of EBV-positive and EBV-negative patients (Figure 1).

The level of both metalloproteinases was significantly higher in patients with DM2 history longer than 10 years (*p* = 10^−6^) and in diabetic patients with obesity (*p* = 10^−4^) (Table 2). The level of MMP-3 in patients with DM2 with a history of more than 10 years was 125.7 pg/mL, while the level of MMP-9 in these patients was 2160.9 pg/mL. In patients with high BMI (30.0–39.9), the levels of MMP-3 and MMP-9 were higher (128.6 pg/mL and 2307.5 pg/mL, respectively) than in those with normal BMI (18.5–24.9) (MMP-3—82.3 pg/mL; MMP-9—1303.0 pg/mL) (Table 2).

## 3. Discussion

It is suggested that inflammation is a crucial factor in the pathogenesis of type 2 diabetes [25,26]. EBV is also a proven risk factor for various malignancies [27], but no studies evaluating the relationships between viral infection and diabetes are available.

All over the world, the incidence of diabetes and cancer as diseases of modern civilization is steadily increasing. Modifiable risk factors such as physical activity, diet, alcohol consumption, smoking, and non-modifiable factors such as age, gender, race, and ethnicity are similar for both diseases. There are reports of more frequent development of malignant neoplasms in diabetic patients, especially in people with DM2 [28]. There is also a correlation between obesity and the development of DM2, cardiovascular disease, and liver cancer [29].

Despite the abundance of data on the possible relationship between MMPs and cardiovascular disease and the widespread knowledge of a significant increase in the incidence and prevalence of cardiovascular disease in people with DM2, the role of MMPs in the development of both micro- and macrovascular complications has only recently begun to be analyzed. Current literature data on this subject are contradictory, and some studies report elevated blood levels of MMPs in people with DM2 [30,31], while some studies have shown no difference in plasma levels of MMPs between people with type 2 DM and without DM [16] or lower concentrations of MMPs in patients with DM2 [32].

Many researchers suggest that MMPs may be associated with the development of obesity as they modulate the adipogenesis process. In obese people with concomitant hypertension, higher levels of MMP-9 were found compared to obese normotensive patients [33,34]. A positive correlation was found between MMP-9 and BMI [35]. The studies of Deros et al. [35] found significantly higher levels of MMP-9 in obese patients compared to those with a normal BMI.

In our research, we observed increased levels of MMPs. The levels of MMP-3 and MMP-9 increased along with higher BMI values in diabetic patients. Our present study also indicated a correlation between the level of MMP-3 and MMP-9 and the duration of diabetes. The level of both metalloproteinases was significantly higher in patients with a DM2 history longer than 10 years (MMP-3 = 125.7 pg/mL; MMP-9 = 2307.5 pg/mL) and in diabetic patients with obesity (MMP-3 = 128.6 pg/mL; MMP-9 = 2160.9 pg/mL).

Some studies indicate increases in the activity of MMPs in various pathological processes [36,37]. Studies by Lin et al. [38] on viral infections and MMPs show that, e.g., HIV infection and HIV/HCV coinfection were identified to increase TIMP1 expression and suppress MMP-3 expression in hepatoma and hepatic stellate cell lines. In addition, MMP-3 has been shown to promote cellular antiviral response against Dengue virus infection [39]. These studies suggest the connection between circulating MMP-3 levels and viral infection may be more complicated than we already know [22,40].

Some researchers, such as Li et al. [40], investigated the expression and activity of MMPs in EBV-positive cell lines. In their study, the EBV-positive C666 cell lines show higher expression and activity of MMP-3 than the EBV-negative cell lines. These findings suggested that infection of EBV may increase the expression of MMP-3 in cells. These results are consistent with the study by Lan et al. [22] that showed that MMP-3 was up-regulated by EBV Zta. The results of the research described above are similar to our results in terms of the increase in metalloproteinase levels due to viral infection. In our study, we compared the levels of MMP-3 and MMP-9 in diabetic patients with EBV infection and without EBV infection. Higher levels of MMP-3 and MMP-9 were observed in diabetic patients with EBV infection. There seems to be a possible association between EBV viral infection and increased levels of metalloproteinases. To our knowledge, there are few publications in the literature on the correlation between viral infection in diabetic patients and the level of metalloproteinases, so further research is needed.

The limitation of our research is a relatively small group of patients. However, the presented studies showed a significant difference in the concentration of both MMPs in diabetic patients additionally infected with EBV compared to the group of non-infected individuals. Therefore, they can modify various metabolic pathways. Further research is needed to evaluate the effects of EBV on various biomarkers that may be important in the diagnosis, prognosis, and monitoring of diabetes mellitus.

## 4. Materials and Methods

### 4.1. Patients

The present study involved 115 patients with DM2 hospitalized in the Internal Ward of the Masovian Specialist Hospital in Radom, Poland. The patients were divided into two groups: EBV-positive and EBV-negative, in which there were 55 and 60 patients, respectively.

### 4.2. Clinical Specimens

Blood and saliva were collected from all patients. EBV DNA was detected in the saliva.

### 4.3. Saliva Collection

About 5 mL of non-stimulated whole saliva was collected. The saliva samples were centrifuged at 1500 rpm at room temperature for 10 min, and then diluted (1:1) in PBS and frozen at −80 °C until their analysis.

### 4.4. Serum Collection

Venous blood samples from all the patients were centrifuged at 1500 rpm at room temperature for 15 min, followed by serum collection, and frozen at −80 °C until their analysis.

### 4.5. Molecular Methods

#### 4.5.1. DNA Extraction from Saliva

DNA isolation was performed using the QIAamp DNA Mini Kit (Qiagen, Hilden, Germany) according to the manufacturer’s instructions. The efficiency and purity of the obtained eluate were checked using the Epoch (Biotek) spectrophotometer. The measurement was performed on a Take 3 plate (Biotek Instruments, Winooski, VT, USA) using Microplate Reader Software Gen 5.2.0 (Biotek Instruments, Winooski, VT, USA).

#### 4.5.2. EBV DNA Detection

EBV DNA detection and the amplification of the Epstein–Barr nuclear antigen 2 (EBNA-2) gene (the nested PCR) were performed as previously described [41]. The nested PCR was carried out for amplification of Epstein–Barr nuclear antigen 2 (EBNA-2). The sequence of primers used for PCR was as follows: outer pair 5′–TTT CAC CAA TAC ATG ACC C–3′, 5′–TGG CAA AGT GCT GAG AGC AA–3′ and inner pair 5′–CAA TAC ATG AAC CRG AGT CC–3′, 5′–AAG TGC TGA GAG CAA GGC MC–3′.

All PCR reactions were carried out in the final volume of 25 μL using HotStartTaq DNA Polymerase (Qiagen, Germany). Concentrations of PCR reaction components were prepared as follows: 2.0 mM MgCl_2_, 0.2 mMdNTPs, 0.5 μM of each forward and reverse primers, and 0.5 U of HotStartTaq polymerase. During each run, the samples were tested together with one negative (nuclease-free water) and positive control (EBV-positive cell line, Namalwa, ATCC-CRL-1432) [42].

#### 4.5.3. MMP-3 and MMP-9

The levels of MMPs in serum were determined by the ELISA method using kits from the CLOUD-CLONE CORP (MMP-3: SEA101Hu and MMP-9: SCA553Hu) according to the manufacturer’s instructions (centrifuge 1000× *g*, dilution of samples: MMP-3 1:100 and MMP-9 1:200). Calibration curves were drawn for each set of the attached standards. Absorbance was read at 450 nm with an Epoch (Biotek) spectrophotometer and converted to numerical values [43].

### 4.6. Statistical Analysis

Statistical analysis was performed using Pearson’s chi-square test, Mann–Whitney U Test and ANOVA Kruskal–Wallis test. The statistical significance was defined as *p* < 0.05.

### 4.7. Ethics

The study was approved by the Medical University of Lublin Ethics Committee, and conforms to GCP regulations (no. KE-0254/121/2021, 27 May 2021). Informed written consent was collected from all participants.

## 5. Conclusions

The presented research showed a significant difference in the concentration of MMP-3 and MMP-9 in diabetic patients additionally infected with EBV compared to the group of non-infected individuals. The level of both MMPs was significantly higher in diabetic patients with EBV-positive and in patients with a diabetic history of over 10 years, as well as in obese patients. Future studies are needed to carefully evaluate the effect of viral infection in diabetic patients on MMPs levels. It seems that MMPs may be useful biomarkers in the diagnosis, prognosis, and monitoring of diabetes associated with EBV infection.

## Figures and Tables

**Table 1:** Epidemiological characteristics of EBV-positive and EBV-negative diabetic patients.

	EBV-Positive N = 55		EBV-Negative N = 60		*p*
	N	%	N	%	
**Sex**					
**Male**	23	41.8	24	40.0	>0.05
**Female**	32	58.2	36	60.0	
**Age**					
**20–39**	7	12.7	8	13.3	>0.05
**40–59**	20	36.4	22	36.7	
**60+**	28	50.9	30	50.0	
**Place of residence**					
**Urban**	23	41.8	24	40.0	>0.05
**Rural**	32	58.2	36	60.0	
**Smoking**					
**Yes**	34	61.8	38	63.3	>0.05
**No**	21	38.2	22	36.7	
**Alcohol abuse**					
**Yes**	35	63.6	36	60.0	>0.05
**No**	20	36.4	24	40.0	
**BMI**					
**18.5–24.9**	9	16.4	11	18.3	>0.05
**25–29.9**	14	25.5	16	26.7	
**30–39.9**	32	58.1	33	55.0	
**Duration of diabetes (years)**					
**1–5**	11	20.0	13	21.7	>0.05
**6–10**	14	25.5	15	25.0	
**>10**	30	54.5	32	53.3	

Duration of diabetes (years)—duration from the time of diagnosis as diabetic; BMI—body mass index, N—number of patients (Pearson’s chi-square test).

**Table 2:** The levels of MMP-3 and MMP-9 compared to BMI and duration of diabetes in the serum of EBV-positive diabetic patients.

BMI	MMP-3 [pg/mL]	MMP-9 [pg/mL]
18.5–24.9	82.3 +/− 5.9	1303.0 +/− 128.7
25.0–29.9	108.5 +/− 16.3	1741.5 +/− 255.2
30.0–39.9	128.6 +/− 2.0	2307.5 +/− 240.9
*p* value *	10^−4^	10^−4^
**Duration of diabetes (years)**		
6–10	90.3 +/− 13.7	1440.4 +/− 226.5
>10	125.7 +/− 4.3	2160.9 +/− 258.4
*p* value **	10^−6^	10^−6^

* Kruskal–Wallis Test, MMP-3 BMI (H = 39.4573), MMP-9 BMI (H = 39.8883); ** Mann–Whitney U Test, MMP-3 Z = 6.0438, MMP-9 Z = 6.172.

## Data Availability

The data presented in this study are available in the article.
